# Case Report: Pyogranulomatous Panniculitis Mimicking an Invasive Subcutaneous Mass with Muscular Pseudo-Invasion in a Dog

**DOI:** 10.3390/vetsci13030232

**Published:** 2026-02-28

**Authors:** Changmin Park, Bumgyu Shin, Sangyul Lee, Hyung-Seok Seo, Jung-Moon Kim, Hwi-Yool Kim

**Affiliations:** Department of Veterinary Surgery, College of Veterinary Medicine, Konkuk University, Neungdongro 120, Seoul 05029, Republic of Korea; cmpark9575@konkuk.ac.kr (C.P.); purple090@konkuk.ac.kr (B.S.); sangyul7918@gmail.com (S.L.); gudtjrdldl@konkuk.ac.kr (H.-S.S.); kimjungm418@gmail.com (J.-M.K.)

**Keywords:** panniculitis, mass, dog, pseudo-invasion

## Abstract

This report describes an atypical presentation of pyogranulomatous panniculitis and perifolliculitis in an 11-year-old mixed-breed dog. The patient presented with a firm, warm cervical mass that progressed with unusual aggression over a matter of days. While advanced diagnostics, including Computed Tomography (CT) and Ultrasonography (US), provided critical data regarding the lesion’s extent, they proved deceptive in determining its etiology; the imaging demonstrated apparent continuity between the mass and the adjacent musculature, strongly favoring a diagnosis of invasive neoplasia. Due to this diagnostic ambiguity and the mass’s rapid expansion, the lesion was surgically excised for definitive analysis. Surprisingly, histopathological examination contradicted the clinical and radiographic suspicion of malignancy, identifying instead a severe multifocal-to-coalescing inflammatory process. The patient recovered rapidly postoperatively with no evidence of recurrence. This case highlights that while imaging is an invaluable tool for surgical planning, it cannot reliably differentiate between aggressive inflammatory processes and invasive masses. The patient’s rapid recovery with no recurrence reinforces this limitation. Consequently, histopathological examination remains the gold standard for definitively diagnosing and guiding the management of rapidly expanding lesions.

## Simple Summary

Pyogranulomatous panniculitis is a condition where the layer of fat just under a dog’s skin becomes severely inflamed. It causes deep, painful lumps to form, which eventually break open and leak a heavy, oily fluid. We are reporting a case of severe pyogranulomatous panniculitis in an 11-year-old mixed-breed dog, characterized by an atypically aggressive mass in the left cervical region that developed over several days. Due to the lesion’s rapid growth and firm consistency, a malignant neoplasm could not be ruled out during the initial physical examination. Diagnostic imaging, including Ultrasonography (US) and Computed Tomography (CT), proved inconclusive; however, the findings indicated continuity between the mass and the adjacent musculature, further suggesting an invasive tumor. Consequently, the lesion was surgically excised for histopathological evaluation, which ultimately revealed severe multifocal-to-coalescing pyogranulomatous panniculitis and perifolliculitis. This case underscores that inflammatory panniculitis can clinically and radiographically mimic the behavior of an invasive malignancy, necessitating prompt surgical intervention and histopathology to differentiate between inflammatory and neoplastic processes.

## 1. Introduction

Panniculitis encompasses a diverse group of inflammatory disorders specifically targeting the subcutaneous adipose tissue [1]. In veterinary medicine, sterile panniculitis typically manifests through a pyogranulomatous inflammatory profile [2]. This is characterized histologically by a mixed infiltrate of neutrophils and macrophages, frequently appearing alongside fat necrosis and multinucleated giant cells, despite a lack of detectable infectious organisms [2]. Clinical observation suggests this condition exists within the broader spectrum of sterile pyogranulomatous dermatitis and panniculitis, an idiopathic or immune-mediated syndrome documented in dogs [3]. Because inflammatory processes in the subcutis can migrate along follicular units, patients may also exhibit concurrent perifolliculitis, where inflammatory cells infiltrate the area surrounding hair follicles in the dermis [4]. Consequently, the simultaneous presence of panniculitis and perifolliculitis is viewed as a single, continuous inflammatory process bridging the dermis and subcutaneous layers, rather than two distinct disease states [3,4].

In veterinary medicine, panniculitis can be diagnosed in several ways with a single tool or multiple tools. Fine Needle Aspiration (FNA) or biopsy is commonly used as a diagnostic tool for panniculitis [5,6]. FNA is frequently employed as an initial diagnostic modality; however, cytologic results may be inconclusive in cases characterized by extensive necrosis, mixed inflammatory infiltrates, or deep tissue involvement [5]. Advanced imaging modalities such as CT and US are not routinely utilized in the diagnostic work-up of panniculitis [5,6]. However, CT and US were carried out in this particular case to assist in the diagnosis of panniculitis.

Idiopathic sterile nodular panniculitis (SNP) in dogs typically presents as multiple subcutaneous nodules or masses ranging in size and distribution. The lesion usually grows relatively slowly, taking several weeks to a few months for the owner to realize [6]. In contrast, rapidly enlarging solitary subcutaneous masses, particularly those exceeding several centimeters in diameter, are more commonly associated with soft-tissue neoplasia in dogs. Such features may complicate the differentiation between inflammatory and neoplastic processes during initial clinical and imaging evaluation [7,8].

SNP is typically defined as a multifocal inflammatory condition of the subcutaneous adipose tissue that frequently extends superficially into the deep dermis, often resulting in draining tracts or ulceration [9]. While the involvement of the panniculus and dermis is well-characterized, clinical continuity with the underlying skeletal musculature is not a standard feature typically emphasized in small-breed presentations [8]. In contrast, deep adherence or fixation to underlying muscle fascia is a hallmark more commonly associated with invasive soft tissue neoplasia, such as sarcomas, rather than inflammatory conditions [7,10]. However, in rare, fulminant cases of SNP, inflammation has been shown to extend into adjacent subcutaneous muscles, causing necrosis and fragmentation of muscle fibers. Such deep continuity was observed in the present case, complicating the initial differentiation between a neoplastic and benign process [8].

Clinically, SNP is widely regarded as an immune-mediated disorder, for which the therapeutic standard of care involves systemic immunosuppression [11]. While this medical approach is effective for inducing remission in multifocal disease, it necessitates long-term management and carries the risk of significant systemic adverse effects. Consequently, surgical intervention remains a critical treatment modality, particularly for solitary lesions or cases where the clinical presentation—such as rapid growth or fixation—mimics soft tissue neoplasia [1,2,12].

This case report describes a unique presentation of solitary pyogranulomatous panniculitis and perifolliculitis that clinically and radiographically mimicked an invasive subcutaneous mass. Unlike typical cases of sterile nodular panniculitis, which are often confined to the subcutis, this lesion exhibited rapid growth and deep muscular adhesion on CT, strongly suggesting malignancy. The purpose of this report is to document this diagnostic challenge and demonstrate that wide surgical excision—while typically reserved for neoplasia—can provide a curative outcome for atypical, tumor-like inflammatory lesions.

## 2. Case Description

**Figure 1:**
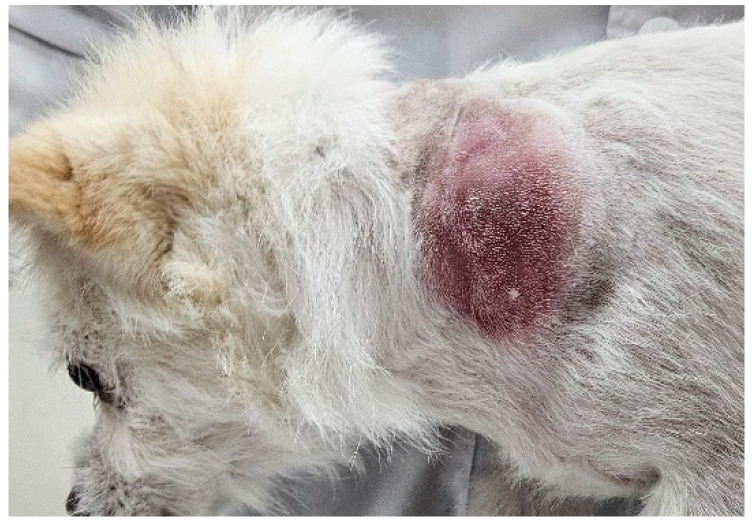
Lateral view of the left neck (C4–C7 level) revealing a large, well-circumscribed, erythematous, and subcutaneous mass.

A 5 kg, 11-year-old mongrel dog (BCS 4/9) was referred to the KonKuk University VMTH presenting a large solitary mass (35.6 mm × 57.3 mm × 48.0 mm) on the left C4–C7 cervical region (Figure 1).

**Figure 2:**
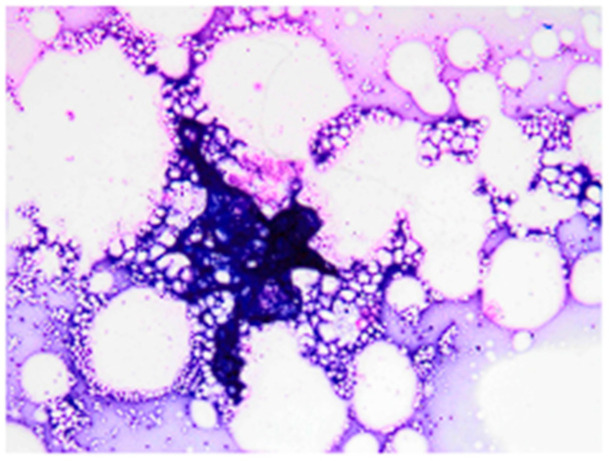
Cytologic evaluation of the fine-needle aspirate (FNA) obtained from the left cervical mass at initial presentation. The sample exhibits low cellularity, characterized predominantly by abundant clear lipid vacuoles and scant macrophages. (Diff-Quik stain, original magnification × 1000).

Following initial detection, the owner reported rapid and aggressive expansion of the lesion over a period of only three days. At presentation, the mass measured 35.6 mm × 57.3 mm × 48.0 mm (Figure 1) and was associated with erythema and localized warmth. Based on the rapid growth rate and gross appearance, the lesion was clinically indistinguishable from aggressive neoplasm. Initial diagnostic evaluation included a complete blood count (CBC) and serum biochemistry, which revealed no clinically significant abnormalities to the chief complaint. FNA of the mass yielded a low-cellularity sample characterized by abundant lipid vacuoles and scant macrophages, consistent with a non-diagnostic or benign fatty proliferation (Figure 2).

**Figure 3:**
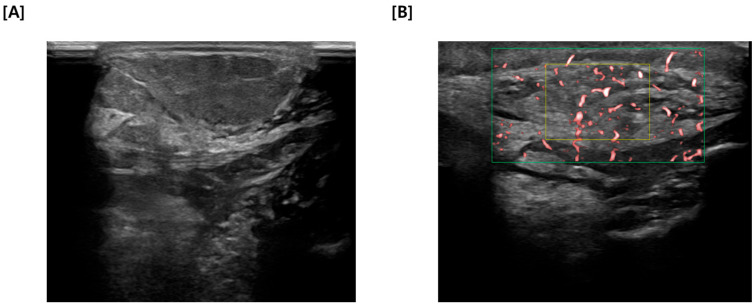
(**A**) Ultrasonographic image of the mass revealing an irregularly marginated structure with heterogeneous echotexture. Note the presence of focal fluid accumulations and hyperechoic areas within the lesion, (**B**) Color Doppler Ultrasound interrogation of the mass revealing distinct internal vascularity. The presence of multiple chaotic blood flow signals (red foci) confirms the tissue is solid and metabolically active, a finding consistent with neoplasia or active inflammation.

Given the incongruity between the cytological results and the clinical presentation, sequential US and CT imaging was warranted. US findings were an irregularly marginated mass with heterogenous echotexture, containing focal fluid and hyperechoic areas (Figure 3A). Figure 3B illustrates the application of color flow Doppler to the identified mass, revealing distinct evidence of intralesional vascularity. The presence of multiple, irregular internal flow signals confirms that the tissue is perfused and metabolically active, rather than an avascular cyst or necrotic accumulation. This pattern of neovascularization was significant because it indicates the recruitment of blood supply often associated with solid mass or active inflammation.

**Figure 4:**
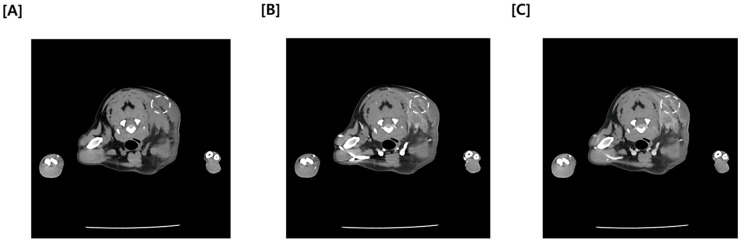
Sequential transverse Computed Tomography (CT) images illustrating multiphase contrast dynamics. (**A**) Pre-contrast phase, (**B**) arterial phase, (**C**) delayed phase. The white dotted circles demarcate an identical region of interest measured across all three images. Note that while the anatomical location remains constant, the mean attenuation value within the circle changes according to the phase, demonstrating progressive enhancement (Pre: 18.46 HU, AP: 52.08 HU, DP: 61.14 HU).

**Figure 5:**
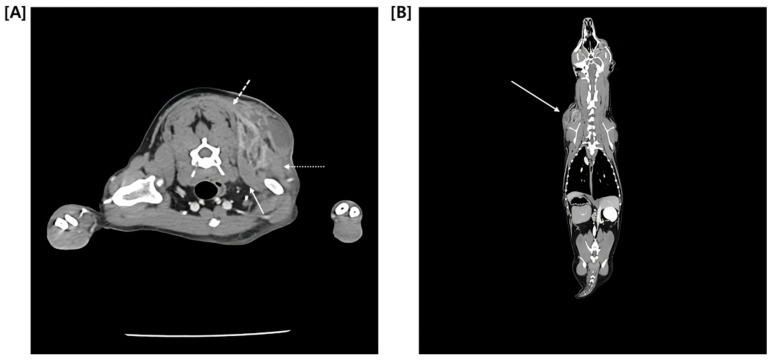
Computed Tomography (CT) findings of the mass. (**A**) Axial image confirming continuity between the medial aspect of the mass and adjacent muscles, including the cranial portion of the trapezius (dotted white arrow), omotransversarius (asterisk-style white arrow), and supraspinatus muscles (white arrow), (**B**) coronal plane image demonstrating the location and extent of the mass (white arrow).

**Figure 6:**
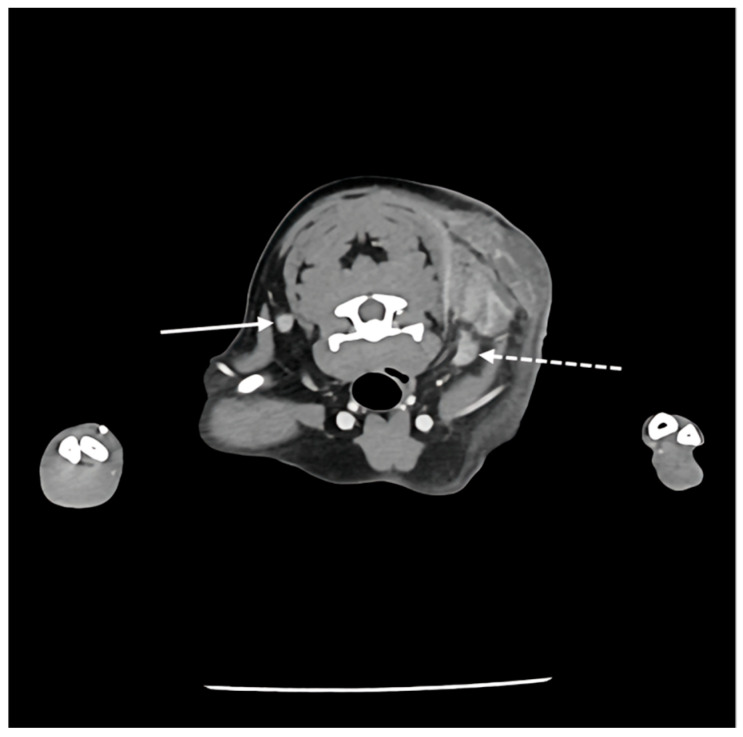
Transverse post-contrast CT image at the level of the cervical lymph nodes. The solid arrow indicates the right prescapular lymph node (4.55 mm), while the dashed arrow indicates the left prescapular lymph node (5.69 mm).

Since Ultrasonography could not definitely exclude the possibility of a malignant tumor, a multiphase CT study comprising pre-contrast, arterial phase (AP), and delayed phase (DP) was promptly performed. Quantitative analysis of the mass revealed relatively low average attenuation values in the contrast-enhanced phase (Pre: 18.46 HU, AP: 52.08 HU, DP: 61.14 HU) (Figure 4). Furthermore, CT findings confirmed continuity between the medial aspect of the mass and adjacent muscles, including the cranial portion of the trapezius muscle, as well as the omotransversarius and supraspinatus muscles (Figure 5). Regarding regional staging, the left prescapular lymph node was noted to be slightly enlarged (Rt: 4.55 mm, Lt: 5.69 mm). Aside from a slight enlargement of the left prescapular lymph node relative to the contralateral side, no significant lymphadenopathy or evidence of metastasis was observed in the regional lymph node (Figure 6).

**Figure 7:**
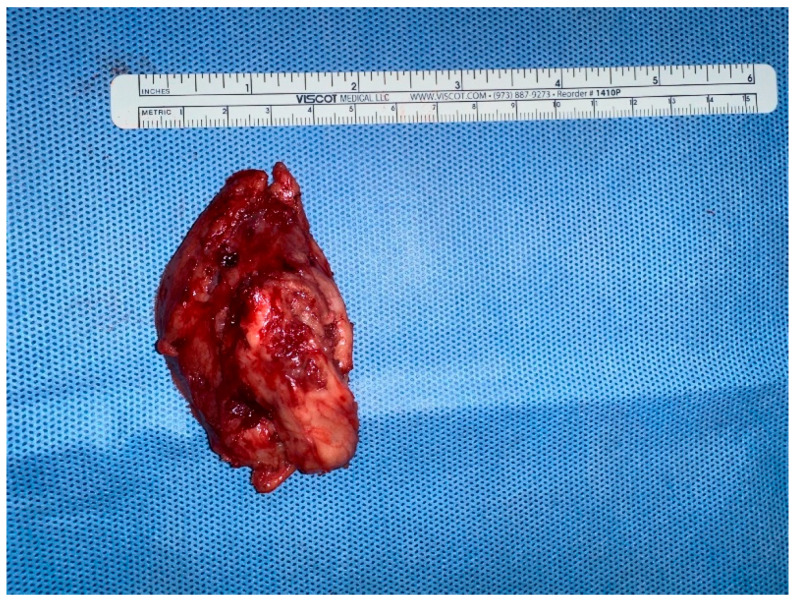
Gross appearance of the surgically excised mass.

Based on the multimodal imaging findings and the rapid progression of the lesion, wide surgical excision was performed to facilitate a definitive diagnosis via histopathology and to ensure complete removal of the suspected malignancy. Due to anatomical constraints in the cervical region, lateral margins of approximately 1–2 cm were obtained; however, to address the suspected skeletal muscle invasion, the deep margin included the underlying muscle fascia and a cuff of the adherent musculature, ensuring complete removal of the suspected neoplastic tract. The lesion was excised and sent for histopathological examination (Figure 7).

**Figure 8:**
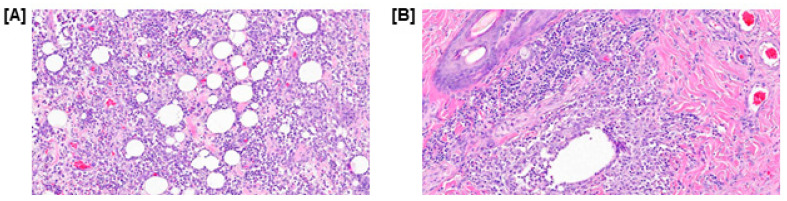
Histopathological evaluation of the excised tissue. (**A**) Section of the subcutaneous adipose tissue demonstrating severe pyogranulomatous panniculitis. Note the extensive infiltration of neutrophils, macrophages, and lymphocytes replacing and surrounding the adipocytes, (**B**) Section of the dermis revealing moderate perifolliculitis, characterized by inflammatory cell accumulation around the hair follicle without evidence of follicular rupture.

The diagnosis of the histopathological examination was severe multifocal to coalescing pyogranulomatous panniculitis and perifolliculitis. Histopathological examination revealed severe pyogranulomatous panniculitis accompanied by moderate dermal perifolliculitis. The subcutaneous tissue presented with multifocal, widely distributed nodules, some exhibiting partial coalescence. Microscopically, these nodules consisted of central neutrophilic aggregates surrounded by macrophages, lymphocytes, and plasma cells, separated by marked fibrosis. While moderate to marked periadnexal inflammation was noted, there was no evidence of follicular rupture, intrafollicular organisms, or neoplastic changes (Figure 8). Also, tissue samples from the excised mass were submitted for aerobic, anaerobic, and fungal cultures; all returned negative results, definitively ruling out infectious etiologies and confirming the sterile nature of the inflammation.

The patient remained hospitalized for three days and was discharged on postoperative day (POD) 4. Postoperative wound management consisted of daily disinfection using 0.05% chlorhexidine and 10% povidone-iodine. No clinical signs of recurrence were observed at the follow-up examination.

## 3. Discussion

While CT is critical for mapping the extent of subcutaneous masses, this case demonstrates that it cannot always reliably distinguish between severe inflammation and neoplasia [13]. In this patient, the CT images provided excellent anatomical detail, but the lesion’s characteristics—specifically its irregular margins, heterogeneity, and contrast enhancement—were virtually identical to those seen in malignant soft tissue tumors [13,14]. In veterinary radiology, indistinct boundaries and evidence of local infiltration are typically considered red flags for soft tissue sarcomas [13]. Furthermore, quantitative analysis of the mass revealed progressive but relatively moderate attenuation values (Pre: 18.5 HU, Arterial: 52.1 HU, Delayed: 61.1 HU). While intense contrast enhancement is often anticipated in malignant lesions, the absence of marked hyperattenuation in this case did not preclude a diagnosis of neoplasia. A recent study by Hong et al. (2025) demonstrated that a soft tissue sarcoma (STS) frequently exhibited significantly lower post-contrast attenuation values (median 63.9 HU) compared to other cutaneous malignancies such as mast cell tumors [15]. This finding is supported by physiological data indicating that sarcomas often possess lower blood volume and perfusion parameters than carcinomas, resulting in moderate rather than intense enhancement. Therefore, the observed attenuation values in this patient were entirely consistent with the imaging profile of a sarcoma, further complicating the differentiation from an inflammatory process [16]. While previous reports have utilized CT to characterize panniculitis, describing it primarily as diffuse subcutaneous fat stranding, the presentation in this case was far more deceptive. Although perilesional stranding was observed, the dominant feature revealed a solid, mass-like structure with complete loss of fascial planes, mimicking the tomographic features of an invasive sarcoma rather than a typical inflammatory process. This diagnostic confusion was further compounded by the failure of fine-needle aspiration to identify the inflammatory nature of the mass. The unreliability of cytology in this condition is well-documented; Kim et al. (2011) reported that in 70% of sterile panniculitis cases—specifically those presenting as firm masses—cytology revealed pleomorphic mesenchymal cells that were misdiagnosed as neoplasia [5]. This discordance confirms that neither CT findings nor cytology can definitively rule out panniculitis, even when the clinical picture strongly suggests a malignancy [8].

The most deceptive aspect of this case was the apparent continuity between the mass and the underlying cervical musculature. Classically, sterile nodular panniculitis is described as a condition limited to the subcutaneous fat and dermis [8]. However, the CT images here showed a complete loss of fascial planes, implying direct invasion into the cranial trapezius, omotransversarius, and supraspinatus muscles. Such deep tissue fixation is a behavior typically reserved for invasive tumors, not inflammatory skin diseases [17]. There are, however, documented instances where severe, deep panniculitis induces such intense fibrosis that it adheres to adjacent musculature, mimicking neoplastic fixation [8]. In this patient, the extensive fibrosis identified on histopathology likely caused this “pseudoinvasion” effect, biasing the preoperative diagnosis toward cancer [2,8].

The temporal evolution of the lesion further obscured the diagnosis. Sterile nodular panniculitis is historically categorized as a chronic condition, with lesions typically persisting for weeks or months [2]. Post (1983) described a classic presentation in which multiple nodules persisted for over a month despite antibiotic therapy, underscoring the protracted course often associated with this disease [18]. In contrast, this patient’s mass was fulminant, expanding to nearly 6 cm in just three days. This rapid growth rate aligned more closely with the biological behavior of a high-grade neoplasm or an acute abscess rather than a sterile inflammatory process [19]. Given the deviation from the standard “slow-growing” timeline, a conservative “wait-and-see” approach was deemed inappropriate.

Additionally, the relative tumor burden presented a significant diagnostic anomaly. While a direct statistical correlation between patient weight and lesion size has not been established in retrospective studies, the available case literature suggests a trend of proportionality. For instance, reports in small breeds, such as a 4-month-old Toy Poodle, describe nodules typically limited to 2–3 cm in diameter [18]. In contrast, “exceptionally large” panniculitis lesions (exceeding 30 cm) have been documented only in large-breed dogs capable of accommodating such volume, such as the German Shepherd [8]. Consequently, the development of a mass of this magnitude represents a disproportionate physical burden rarely encountered in small-breed patients. This substantial mass effect further supported the clinical suspicion of an aggressive neoplasm rather than a benign inflammatory condition.

Finally, the therapeutic approach was adapted to address both the diagnostic uncertainty and the owner’s urgency. Sterile panniculitis in dogs is most commonly managed medically using systemic immunosuppressive agents such as prednisolone or cyclosporine, particularly in cases with multifocal involvement [5,20]. While the standard medical management for multifocal sterile panniculitis involves systemic immunosuppression with drugs like prednisone or cyclosporine [20], the presumptive diagnosis of malignancy drove the decision toward immediate intervention. Although surgery is typically reserved for solitary or ulcerative lesions, wide excision in this case proved to be both diagnostic and curative [6]. The patient’s complete recovery without recurrence suggests that for solitary, aggressive-appearing panniculitis lesions, surgical resection is a definitive treatment option that spares the patient from the potential side effects of long-term immunosuppressive therapy [6].

This study has limitations inherent to single-case reports. First, because this is a retrospective description of a unique clinical presentation, the specific CT findings of muscle ‘pseudoinvasion’ may not be generalizable to all cases of solitary panniculitis. Second, the lack of an initial therapeutic trial with corticosteroids—owing to the owner’s preference for surgical intervention—prevented the assessment of whether this fulminant lesion would have responded to standard medical management alone. Finally, the follow-up period, while sufficient to confirm surgical recovery, was limited in duration; therefore, the long-term potential for relapse or the development of multifocal lesions elsewhere remains unknown.

## 4. Conclusions

This case demonstrates that sterile pyogranulomatous panniculitis can present with fulminant growth and radiological features indistinguishable from invasive subcutaneous tumors, including indistinct margins and apparent muscular invasion. Clinicians should be aware that CT evidence of deep tissue fixation is not always pathognomonic for neoplasia and that severe inflammation can breach fascial planes in small-breed dogs. Consequently, even when imaging strongly suggests a tumor, panniculitis remains a critical differential diagnosis. In cases of solitary, rapidly expanding lesions where cytology is inconclusive or medical trial is declined, aggressive en bloc excision serves a critical role. It provides the definitive histopathological and microbiological diagnosis required to rule out neoplasia and infection, while simultaneously ensuring a curative outcome by completely resecting the inflammatory tract and its deep muscular attachments, thereby sparing the patient from the adverse effects of long-term immunosuppressive therapy.

## Data Availability

The original contributions presented in this study are included in the article. Further inquiries can be directed to the corresponding author.

## Abbreviations

The following abbreviations are used in this manuscript:

POD	Postoperative days
FNA	Fine Needle Aspiration
SNP	Sterile Nodular Panniculitis
CT	Computed Tomography
US	Ultrasonography
HU	Hounsfield Unit
AP	Arterial Phase
DP	Delayed Phase
